# The Effect of Stress on Working Memory in Persons with Parkinson’s Disease

**DOI:** 10.3390/brainsci16030319

**Published:** 2026-03-17

**Authors:** Andrew Zaman, Caelia Marshall, Elizabeth L. Stegemöller

**Affiliations:** Department of Kinesiology, Iowa State University, Ames, IA 50011, USA; afz20@med.miami.edu (A.Z.); cmarshal@iastate.edu (C.M.)

**Keywords:** Parkinson’s disease, stress, working memory, digit span, cold pressor

## Abstract

Background: In addition to motor symptoms, persons with Parkinson’s disease (PD) experience several non-motor symptoms with challenges in working memory being particularly common. These cognitive challenges may worsen under stress. The purpose of this pilot study was to examine how physical stress affects working memory in persons with PD. Methods: Eight individuals with PD and 11 healthy older adults (HOAs) completed digit span forward and backward tasks following a socially evaluated cold pressor stressor and a control condition. Results: Under non-stressful conditions, persons with PD had a smaller digit span backward capacity and were slower during the digit span forward task compared to HOAs. However, during the stress condition, individuals with PD performed comparably to HOAs on the backward digit span task. Stress negatively affected response times on the backward task for both groups but did not alter capacity or response time on the forward task. Conclusions: These findings provide an initial step in understanding the effects of physical stress on working memory in PD. Since working memory supports many daily activities, understanding how stress influences this cognitive process may inform interventions that enhance stress regulation and improve cognitive and functional outcomes for individuals living with PD.

## 1. Introduction

Persons with Parkinson’s Disease (PD) often experience impairments in cognitive functioning—a strong predictor for quality of life—at the earliest stages of the disease [1,2,3,4]. These impairments are diverse and include changes in attention, executive functions, memory, language, and visuospatial abilities [5]. Some estimates suggest that up to 80% of individuals with PD will develop dementia [6]. However, executive functions, such as working memory, which are associated with the ability to complete common daily activities, appear to be the most negatively affected cognitive domain in persons with PD [6,7]. As such, persons with PD demonstrate working memory deficits [8,9,10,11,12] as well as processing impairments in in frontal lobe areas [6,12,13]. There remains a need to better understand how common life factors, such as stress, influence impairments in working memory in persons with PD.

Research has shown that stress negatively impacts working memory in healthy populations, particularly during tasks that rely on prefrontal cortex functioning [14,15,16]. Meta-analytical investigations suggest that stress negatively affects working memory tasks by creating higher working memory loads [17]. One possible explanation for this effect may be that stress leads to excess catecholamines, dopamine, and norepinephrine in the prefrontal cortex, which disrupt the balance of dopaminergic activity necessary for optimal working memory function [14,18,19,20,21,22]. Medications including dopaminergic agonists and precursors, which are commonly used to ameliorate motor symptoms in persons with PD, may unintentionally exacerbate the detrimental effects of excess dopamine in prefrontal regions [23]. Given the relationships among stress, dopamine, and working memory in healthy populations, it is likely that stress may further impair working memory performance in persons with PD, who already experience dopaminergic dysfunction. However, the effects of stress on working memory in persons with PD remain largely under explored. Thus, the purpose of this pilot study is to examine the effects of an acute stressor (socially evaluated cold pressor (SECP) [24]) on a simple working memory task (digit span forward and backward) in persons with PD and healthy older adults (HOAs). We hypothesized that stress will negatively impact working memory capacity (digit span forward and backward two error maximum length (TE-ML)) in both HOAs and persons with PD. We also hypothesized that persons with PD would demonstrate lower working memory capacity overall, compared to HOAs.

## 2. Materials and Methods

### 2.1. Recruitment

Eight participants diagnosed with idiopathic PD (mean age 67.8 ± 4.7 years; 5 males and 10 females) and 11 age- and gender-matched HOAs (mean age 68.7 ± 5.0 years) completed this pilot study. The primary inclusion criterion was between the ages of 50 to 80. Participants were excluded from the pilot study for the following reasons: (1) demonstrated cognitive impairment (Mini-Mental Status Exam < 24); (2) severe hearing loss; and (3) systolic blood pressure (SBP) above 140 mmHg or diastolic blood pressure above 90 mmHg during the initial screening. One HOA was excluded during the initial blood pressure screening to avoid any potential cardiac events during the SECP. All participants provided written informed consent. All procedures were approved by the Iowa State University Institutional Review Board.

### 2.2. Procedure

**Figure 1:**
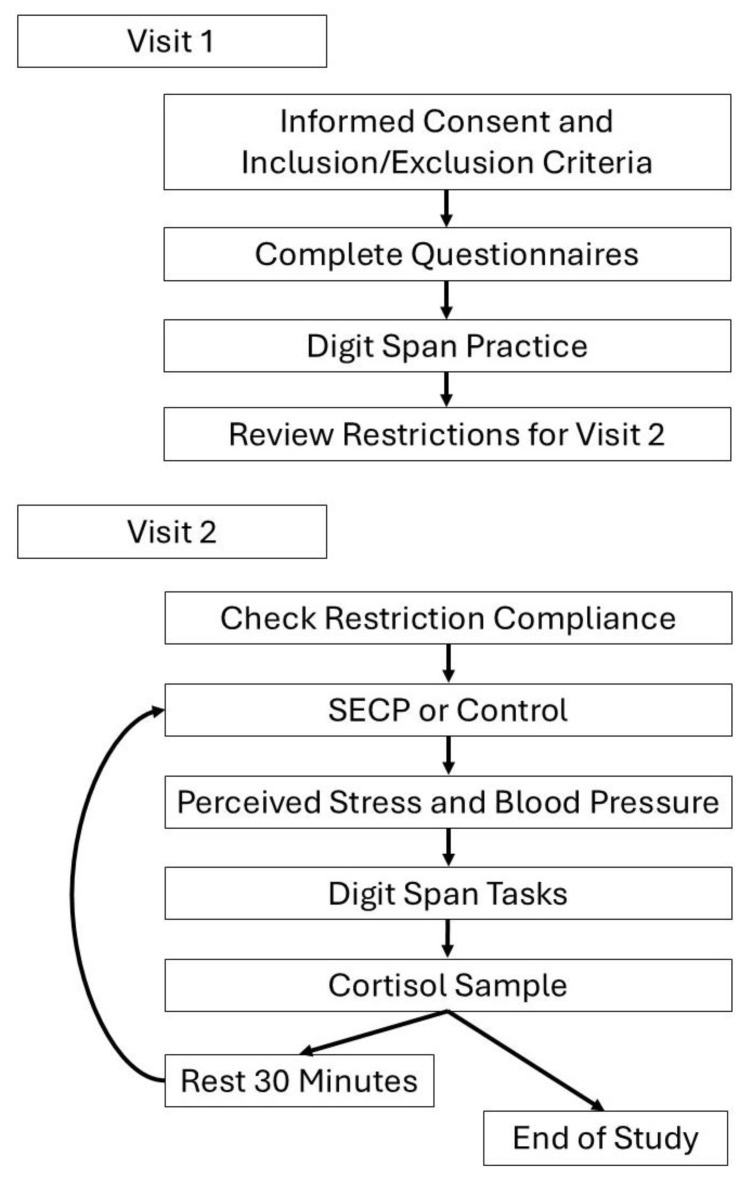
Data Collection Procedure.

Participants engaged in two lab visits within the timespan of one week (Figure 1). The first visit lasted approximately one hour. During the first visit, participants were screened for cognitive impairment with the Mini-Mental Status Exam (MMSE) and high blood pressure using an automated Omron blood pressure cuff. Participants also provided demographic information and completed a battery of reliable questionnaires (Table 1), including the Montreal Cognitive Assessment (MOCA) [25]; Geriatric Depression Scale (GDS) [26]; and the State and Trait Anxiety Inventory (STAI) [27]. The Perceived Stress Scale (PSS) was also collected. While the PSS has not been tested for its psychometric properties in persons with PD, it has been shown to be reliable for use with older adults [28,29]. Participants with PD also completed the Movement Disorders Society-Unified Parkinson’s Disease Rating Scale (MDS-UPDRS) with a trained researcher. See Table 1 for participant demographics and questionnaire results. All participants completed two practice trials of the digit span forward and two practice trials of the digit span backward to familiarize themselves with the working memory tasks during the first visit. Finally, participants were instructed to adhere to dietary and medical restrictions prior to their second lab visit, which included limiting their caffeine intake to one cup (8 oz.) of a caffeinated beverage at least three hours prior to the start of the second visit. Participants were also asked to refrain from alcoholic beverages during the 24 h prior to the start of the second visit. Participants were also asked to eat one to two hours before the visit and to consume their Parkinson’s medication one hour prior to beginning the second visit. The second visit was scheduled to align with each participants’ routine medication times. All second visit start times were scheduled between 12:00 p.m. and 3:00 p.m. Participants were asked to reschedule if they were non-compliant with any of these restrictions.

The second visit, which spanned approximately 2.5 h, began with a review of adherence to dietary and medical restrictions. Baseline collection of salivary cortisol was collected. Participants completed either the stress intervention (SECP) or the control intervention (warm water hand bath). The order was counterbalanced. After completing either, the stress task or the control task, participants’ blood pressures were taken with an automated Omron blood pressure cuff and salivary cortisol was collected. Then, they verbally reported the stress level of the task on a Likert scale from 1 to 10, with respective response anchors of “not stressful at all” and the “most stressful thing I can imagine.’’ Within one to two minutes of completing the stress and control tasks, participants completed the digit span forward and backward tasks. Following these trials, participants rested or read for half an hour. After the rest period, participants repeated the same series of steps and tasks with the uncompleted intervention (i.e., warm water bath or SECP) (Figure 1). Salivary cortisol was collected 25 min after each condition.

### 2.3. Stress and Control Tasks

The stress intervention was conducted via a SECP task where the participants placed their hand up to the wrist in an ice water bath (2–4 °C) for 90 s. Participants were informed that this task included being video recorded to be judged by trained experimenters at a later time. During the control condition, participants placed their hand in warm water (36–38 °C) for 90 s. During the control condition participants were informed that they were not being video recorded and the camera was moved out of sight.

### 2.4. Digit Span Forward and Backward

E-Prime (Psychology Software Tools, Pittsburgh, PA, USA) was used to present the digit span tasks to the participants. Digits were auditorily presented at a rate of 1 per second. The string of digits used in the task did not include the digit 0, sequences (e.g., 1-2, or 2-1) or single digit repetition (e.g., 1, 5, 1) [30]. When beginning the tasks participants were presented with a three-digit number and asked to type the digits back into the computer in the forward or reverse (backward) order. If they got the sequence correct, the number of digits increased by one. Accuracy and response time were recorded via E-Prime. After 2 consecutive mistakes, the task ended. The number of trials correct prior to two successive misses was recorded. This is referred to as the two-error maximum list length (TE-ML) [30]. For correct trials, the response time was the amount of time the participant took to enter the digits back into the computer after the computer completed presenting the digits.

### 2.5. Cortisol Analysis

On the day of collection, salivary cortisol samples were stored in −20 °C freezer within 30 min. Cortisol was analyzed with the Salimetrics^®^ Cortisol Enzyme Immunoassay Kit (Salimetrics, State College, PA, USA). The kit uses a competitive immunoassay in which cortisol competes with cortisol conjugated to horseradish peroxidase for the antibody binding sites. To determine the cortisol reactivity, the area under the curve was calculated using the baseline and the 25 min post intervention samples for each condition.

### 2.6. Statistical Analysis

For all variables, outliers above or below two standard deviations were winsorized [31]. This criterion resulted in 4 of the 106 response time data values of the digit span backwards, 14 of the 178 response time data values of the digit span forwards and 4 of the 60 cortisol data values being winsorized. The normality of the data set was confirmed after winsorization.

An independent samples *t*-test was used to examine any group differences on demographic and questionnaire outcome measures. To confirm that the SECP initiated an increase in stress for participants, a 2 condition (stress, control) × 2 group (PD, HOA) repeated measures ANOVA was completed for measures of stress (perceived stress, blood pressure, and cortisol). To test the hypothesis that stress negatively impacted working memory capacity, and that persons with PD had a lower working memory capacity than HOAs, a 2 condition (stress, control) × 2 group (PD, HOA) repeated measures ANOVA was used to determine differences on digit span forward TE-ML, and digit span backward TE-ML. When examining digit span response time, an additional factor of digit length was also included in the repeated measures ANOVA. For the digit span forward 96.7% of participants successfully completed both trials for the 5 digit length, while only 60% of participants successfully completed the 6-digit length. Thus, digit lengths 3, 4, and 5 were used to examine response time on correct trials using a 2 condition (stress, control) × 2 group (PD, HOA) × 3-digit length (3, 4, 5) repeated measures ANOVA. For the digit span backwards, 93.3% of participants successfully completed both trials for the 3-digit length, 73.3% successfully completed both trials of the 4-digit length, and 50% completed both trials of the 5-digit length. For our analysis digit span backwards response time, a 2 condition (stress, control) × 2 group (PD, HOA) × 2-digit length (3, 4) repeated measures ANOVA was used. For all repeated measures ANOVAs, partial eta squared ($\eta \rho^{2}$) effect sizes were calculated.

Planned post hoc mean comparison *t*-tests were then used for response time measures when analyzing within and between subjects in each intervention at each digit length. Significance was set at α = 0.05. For all post hoc analyses, a Bonferroni correction was used to interpret statistical significance for multiple comparisons.

## 3. Results

### 3.1. Participants

Participants with PD were an average of 67.8 ± 4.7 years and 33.3% were male. HOAs were an average of 68.7 ± 5.0 years and 33.3% male. There were no significant differences in demographic variables between groups. Participants with PD had an average score of 66.9 ± 5.8 on the MDS-UPDRS, were an at an average H&Y stage of 2.3 ± 0.1 and had an average diagnosis duration of 9.9 ± 1.7 years.

For the cognitive questionnaires, participants with PD scored an average of 29.3 ± 0.7 on the MMSE and 26.1 ± 2.2 on the MOCA, HOAs scored an average of 29.7 ± 0.6 on the MMSE and 26.5 ± 2.5 on the MOCA. There were no significant differences between groups on the cognitive measures. For depression and anxiety, participants with PD scored an average of 6.5 ± 4.5 on the GDS and an average of 13.5 ± 5.6 on the PSS. HOAs scored an average of 3.8 ± 3.7 on the GDS and an average of 9.2 ± 6.6 on the PSS. While scores on the GDS and PSS were lower for HOAs, there were no significant differences between groups. For the STAI1 and STAI2, participants with PD scored an average of 33.3 ± 8.5 on the STAI1 and an average of 38.3 ± 8.7 on the STAI2. HOAs score an average of 28 ± 10.9 on the STAI1 and 29.1 ± 7.6 on the STAI2. There were no significant differences between groups for the STAI1. However, there was a significant difference for the STAI2. Participants with PD had higher trait anxiety than the HOAs (*t*_(28)_ = 3.086, *p* = 0.005, Mean Difference (*MD*) = +9.2, *d* = 1.126) (Table 1).

### 3.2. Stress Response

To ensure that the SECP task induced a stress response, perceived stress, blood pressure, and cortisol were measured. All outcome measures revealed an effect of stress (Table 2). For perceived stress, there was a main effect of condition ($F_{\left(1,28\right)}$ = 147.393, *p* < 0.0001, $\eta \rho^{2}$ = 0.840). The participants found that the SECP condition was more stressful than the control condition (*MD* = +5.2, *d* = 2.256). For blood pressure, there was a main effect for condition both systolic ($F_{\left(1,28\right)}$ = 8.003, *p* = 0.009, $\eta \rho^{2}$ = 0.222) and diastolic blood pressure ($F_{\left(1,28\right)}$ = 5.543, *p* = 0.026, $\eta \rho^{2}$ = 0.165). The participants had higher systolic blood pressure after the SECP compared to the control condition (*MD* = +6.4 mmHg, *d* = 0.518). Participants also had higher diastolic blood pressure after the SECP compared to the control condition (*MD* = +2.8 mmHg, *d* = 0.430). Finally, there was a main effect of condition for cortisol (*F*_(1,28)_ = 6.780, *p* = 0.015 $\eta \rho^{2}$ = 0.195). Participants had a higher cortisol AUC value during the SECP condition compared to the control condition (*MD* = +13.4 ng/dL/h, *d* = 0.479). There were no main effects for group (*F*_(1,28)_ < 0.360, *p* > 0.061, $\eta \rho^{2}$ < 0.013), and no interaction effects of group × condition (*F*_(1,28)_ < 0.889, *p* > 0.354, $\eta \rho^{2}$ < 0.031) for all stress related outcome measures.

### 3.3. Digit Span Forward and Backward Two Error Maximum Length

**Figure 2:**
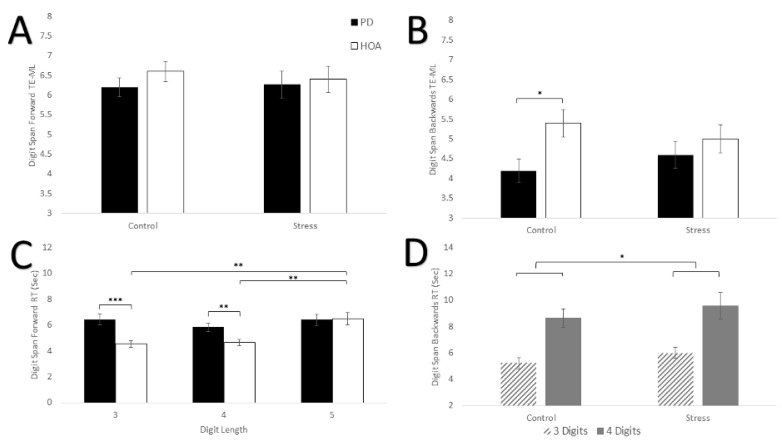
The Effects of Stress on Working Memory. (**A**) Digit span forward TE-ML for both groups during the control and stress conditions. (**B**) Digit span backward TE-ML for both groups during the control and stress conditions. (**C**) Digit span forward response time for both groups with conditions (control, stress) collapsed at each digit length. (**D**) Digit Span Backward response time for the control and stress condition and groups (HOA, PD) collapsed at digit lengths 3 and 4. Standard error bars shown. Small horizontal bars show between group differences. Long horizontal bars show within group differences. Long horizontal bars connected with short horizontal bars indicate a main effect of condition. * *p* < 0.05, ** *p* < 0.01, *** *p* < 0.001.

Table 3, Figure 2A,B shows results for digit span forward and backward TE-ML. Results revealed no differences between participants with PD and HOAs in digit span forward capacity for either condition. Persons with PD did demonstrate a smaller digit span backward capacity that HOAs in the non-stress condition, but this did not reach significance. During the stress condition, digit span backward capacity increased for participants with PD while capacity decreased for HOAs. For the digit span forward TE-ML, there were no main effects of condition, group, or interaction effect (*F*_(1,28)_ < 0.565, *p* > 0.459, $\eta \rho^{2}$ < 0.020). For the digit span backward TE-ML, no main effect for condition or group (*F*_(1,28)_ < 3.632, *p* > 0.067, $\eta \rho^{2}$ < 0.115) was found. In general, HOAs had a larger digit span backward TE-ML than persons with PD (*MD* = 0.8, *d* = 0.617). There was also no interaction effect for group × condition (*F*_(1,28)_ = 3.429, *p* = 0.075, $\eta \rho^{2}$ = 0.109). However, persons with PD increased their digit span backward TE-ML (*MD* = +0.4, *d* = 0.356) score following the SECP, while HOAs demonstrated a decrease (*MD* = −0.4, *d* = 0.322).

### 3.4. Digit Span Forward Response Time

Figure 2C indicated results for digit span forward response time. The SECP task did not impact response time. However, participants with PD had slower response times than HOAs, particularly for the 3-digit and 4-digit sequences. There was no main effect of condition for digit span forward response time (*F*_(1,28)_ = 0.398, *p* = 0.533, $\eta \rho^{2}$ = 0.014). A main effect of group, (*F*_(1,28)_ = 5.579, *p* = 0.025, $\eta \rho^{2}$ = 0.166) was revealed. HOAs had a faster response time compared to persons with PD (*MD* = −0.9 s, *d* = 0.493). There was also a main effect of digit length after applying a Greenhouse-Geisser correction for lack of sphericity (*F*_(1.628,45.575)_ = 12.517, *p* < 0.001, $\eta \rho^{2}$ = 0.309). After applying a Bonferroni correction, post hoc analysis showed that there was a significant difference in response time with a 5-digit length compared to the 3-digit length (*p* = 0.008, *MD* = 1.03 s, *d* = 0.413), and the 4-digit length (*p* < 0.001, *MD* = 1.21 s, *d* = 0.641). There was no difference between the 3-digit length and the 4-digit length (*p* = 1.0, *MD* = 0.18 s, *d* = 0.119).

The results also revealed a length × group interaction effect (*F*_(df = 1.628,45.575)_ = 6.245, *p* = 0.007 $\eta \rho^{2}$ = 0.182). Post hoc analysis showed that there were significant differences between HOAs and persons with PD at the 3-digit length (*p* < 0.001, *MD* = 1.7 s, *d* = 1.346) and 4-digit length (*p* < 0.001, *MD* = 1.1 s, *d* = 1.098), where persons with PD had a slower response time. There was no significant difference at the 5-digit length (*p* = 0.881, *MD* = −0.1 s, *d* = 0.049). Post hoc analysis also revealed that HOAs had a slower response time at the 5-digit length compared to the 3-digit length (*p* < 0.001, *MD* = +1.9 s, *d* = 0.904) and 4-digit length (*p* < 0.001, *MD* = +1.8 s, *d* = 0.802), while there was no difference between the 3- and 4-digit lengths (*p* = 0.654, *MD* = 0.1 s, *d* = 0.082). For persons with PD, post hoc analysis revealed no significant differences among any of the digit lengths. No condition × group, condition × length, or condition × length × group interaction effects were found (*F*_(df = 1.933,54.124)_ < 1.842, *p* > 0.168, $\eta \rho^{2}$ < 0.062).

### 3.5. Digit Span Backward Response Time

Figure 2D shows results for digit span backwards response time. The SECP task slowed response time for the digit span backwards for both groups, but there was no difference in response times between participants with PD and HOAs. There was a main effect of condition (*F*_(1,20)_ = 4.558, *p* = 0.045, $\eta \rho^{2}$ = 0.186) and length (*F*_(1,20)_ = 28.645, *p* < 0.001, $\eta \rho^{2}$ = 0.589). In general, participants had slower response times during the SECP compared to the control condition (*MD* = +0.8 s, *d* = 0.314), and had a slower response times for the 4-digits compared to 3-digits (*MD* = +3.5 s, *d* = 0.984). There was no main effect for group (*F*_(1,20)_ = 0.717, *p* = 0.407, $\eta \rho^{2}$ = 0.035), and there were no interaction effects (*F*_(1,20)_ < 0.434, *p* > 0.518, $\eta \rho^{2}$ < 0.021).

## 4. Discussion

Our results revealed that the SECP intervention was successful in increasing stress, both perceptually and physiologically (perceived stress, blood pressure, and cortisol) in both HOAs and persons with PD. Under non-stressful conditions, persons with PD had a smaller digit span backward capacity and were slower during the digit span forward task (*MD* = +0.9 s) compared to HOAs. However, during the stress condition, persons with PD performed similarly to HOAs on the digit span backward task. Thus, as expected, stress reduced capacity in HOAs (*MD* = −0.4), but surprisingly increased capacity in persons with PD (*MD* = +0.4), equalizing performance. The results also revealed that stress had a negative influence on the amount of time it took to manipulate information response time on the digit span backwards task (*MD* = +0.9 s) in both groups. Stress did not appear to influence digit span forward capacity or response time for either group.

Consistent with much of the previous research in this area and our expected hypothesis, these results showed that persons with PD had smaller working memory capacity on the digit span backward task during the control condition (no stress) [9,10,12]. While some studies have found no differences in digit span backward capacity, this may have been due to testing newly diagnosed persons [32], small sample size [33], and those in the earlier stages of the disease (motor UPDRS = 19.81) [34]. The participants in this pilot study were in the middle stages of the disease (H&Y = 2.3; MDS-UPDRS = 66.9) and thus may be more likely to display lower digit span capacity scores. In contrast, there were no differences between persons with PD and HOAs on the digit span forward task. While a meta-analysis showed that persons with PD have a smaller digit span forward capacity, the effect size of the current pilot study was small (Hedges’ *g* = 0.18) [12], which likely reduced the ability to find any group differences possibly due to being underpowered. Further research with a larger and more diverse sample would aid in further clarifying memory impairment in persons with PD.

Interestingly, the acute stressor equalized performance between the two groups by improving scores in persons with PD and decreasing scores in HOAs. The two currently prevailing theories of why persons with PD have impaired working memory are due to either (1) dopaminergic medication ‘over-dosing’ of prefrontal regions, or (2) reduced striatal dopamine [23,35,36]. Previous literature has shown, though, by inducing acute stress, both prefrontal and striatal dopamine levels presumably increased [37,38,39,40,41]. Thus, the positive effect of stress on working memory capacity in our sample of persons with PD who were in the middle stages of the disease (H&Y = 2.3) may suggest that an increase in prefrontal and striatal dopamine levels due to stress may compensate the reduced striatal dopamine resulting in improved working memory performance after an acute stressor. Indeed, the SECP was a physical stressor, which has been shown to impact dorsal striatal dopamine more than psychological stressors [42]. Future imaging research is needed to test this notion that mild physical stress may compensate the reduced striatal dopamine in persons with PD.

In contrast to the digit span backward task, the current results revealed that physical stress had no effect on the digit span forward task for either persons with PD or HOAs. While a few studies report that stress negatively influences digit span forward capacity [41], most studies find that stress does not influence simple working memory tasks that do not require manipulation of information (i.e., digit span forward) [17,43,44,45]. Overall, the lack of differences between HOAs and persons with PD, and the lack of an effect of the acute physical stressor, may suggest that updating information for simple working memory tasks may be less sensitive to dopaminergic modulation as compared to more complex working memory tasks that require manipulation of information (i.e., digit span backward). Additional research to parse out the underlying physiology of these components of working memory are needed for the development of better therapeutic strategies for cognitive impairment in persons with PD.

Measuring response time of the digit span is an uncommon measure of working memory or other cognitive processes, but it has been employed by some researchers [46,47]. For the digit span task, response time is thought to reflect general processing speed [48]. Thus, the digit span forward response time may be more representative of simple processing speed, as reordering of the digits was unnecessary. Other studies find that stress improves response time on working memory tasks such as the Sternberg working memory task in healthy adults [49]. In this pilot study, HOAs had a quicker response time on the digit span forward during the stress condition, which suggests that stress improved processing speed for this group. Interestingly, persons with PD did not see this same decrease in response time following the SECP stressor. One possible explanation for this is that stress may have negatively impacted PD motor symptoms and how quickly persons with PD were able to reenter the digits on the keyboard. While the literature on how physical stress affects PD motor symptoms is limited, stress has been shown to negatively affect skilled reaching in PD rat models [50]. Thus, more studies are needed to disentangle the effects stress has on motor symptoms, processing speed, and working memory in persons with PD.

For the digit span backward task, response time would include both the time needed to reenter the digits as well as the time needed to reorder the digits. In our pilot study, the results showed that physical stress had a negative impact on response time in both HOAs and persons with PD. These consistent findings for both groups, and the opposite effect in HOAs during the forward digit span (i.e., quicker response time), may suggest that stress negatively influenced the time needed to manipulate/reorder the digits. Thus, while physical stress may have improved working memory capacity in persons with PD, it may have hindered the speed at which they were able to manipulate the information. Another possibility is that physical stress improved the speed of both general processing and manipulating information in persons with PD, but improvements were masked by the worsening of motor symptoms and impairment in fine motor skills. While this pilot study is among the few to investigate the influence of stress on working memory in PD, the results of this pilot study should be cautiously interpreted due to the low number of participants. Future studies that use a different response measure (e.g., something not reliant on motor skills) are needed to separate motor and cognitive components to better understand how stress affects response time in persons with PD.

### Limitations

A major limitation of this pilot study was the small sample size and consequent increased risk for Type II errors. While there was a statistical trend for improvements in persons with PD following stress and decrements in HOAs, this may have been significant with a larger sample. Similarly, no differences between HOAs and persons with PD on the digit span forward capacity were found. With a larger sample size, there may have been group differences and an interaction similar to what was seen in the digit span backwards. Another major limitation was using the keyboard, while this is unlikely to affect TE-ML measures, it could have had differential effects on the response times of persons with PD. Response times may have been inflated in persons with PD compared to processing/cognitive speed due to PD motor symptoms such as bradykinesia (slowness of movement). Additional studies using verbal reports of digits which may be less affected by bradykinesia may be a better indicator of processing/cognitive speed in persons with PD. Finally, dopamine and other neurotransmitters were not measured in this pilot study, which will be needed to confidently conclude the mechanisms involved in the influence of stress on working memory in persons with PD.

## 5. Conclusions

Taken together, these results suggest that physical stress does not have a universally negative effect on persons with PD. While the underlying mechanisms were not examined in this pilot study, the results may suggest that improvement in working memory capacity may be mediated by increases in striatal dopamine due to the stress response. Results also demonstrated that physical stress may have improved simple processing speed in HOAs, but remains unclear in persons with PD due to the potential confound of motor impairment. Overall, this pilot study showed that a mild physical stress may improve working memory in persons with mild to moderate PD. The results of this pilot study provide initial evidence for future needed studies to better understand the complex impact of stress on symptoms of PD.

## Figures and Tables

**Table 1:** Demographic and Questionnaire Information.

	PD	HOA
Age (Years)	67.8 ± 4.7	68.7 ± 5.0
Gender (% Male)	33.3 ± 50.0	33.3 ± 50.0
MDS-UPDRS	66.9 ± 5.8	N/A
H&Y	2.3 ± 0.1	N/A
Years since diagnosis	9.9 ± 1.7	N/A
MMSE	29.3 ± 0.7	29.7 ± 0.6
MOCA	26.1 ± 2.2	26.5 ± 2.5
GDS	6.5 ± 4.5	3.8 ± 3.7
PSS	13.5 ± 5.6	9.2 ± 6.6
STAI1	33.3 ± 8.5	28 ± 10.9
STAI2	38.3 ± 8.7 **	29.1 ± 7.6 **

Means and standard deviations for all demographics and questionnaires for both groups. For a main effect of group ** *p* < 0.01. HOA; healthy older adult; PD: persons with Parkinson’s disease; MDS-UPDRS: Movement Disorders Society-Unified Parkinson’s Disease Rating Scale; H&Y: Hoehn & Yahr; N/A: Measure specific to Parkinson’s disease; MMSE: Mini Mental State Exam; MOCA: Montreal Cognitive Assessment; GDS: Geriatric Depression Scale; PSS: Perceived Stress Scale; STAI: State and Trait Anxiety Inventory.

**Table 2:** Results of Stress Test.

	PD Control	HOA Control	PD Stress	HOA Stress
Perceived Stress ***	1.20 ± 0.56	1.00 ± 0.00	6.40 ± 2.53	6.13 ± 2.27
SBP (mmHg) **	121.3 ± 13.0	130.5 ± 16.2	129.7 ± 15.4	134.7 ± 13.8
DBP (mmHg) *	75.6 ± 9.3	74.9 ± 8.0	79.5 ± 8.7	76.7 ± 8.3
Cortisol (ng/dL/h) *	66.7 ± 26.4	58.8 ± 23.2	76.1 ± 27.0	76.3 ± 34.2

Means and standard deviations for all stress measures for both groups and both conditions. For a main effect of condition * *p* < 0.05, ** *p* < 0.001, *** *p* < 0.0001. HOA; healthy older adult; PD: persons with Parkinson’s disease; DBP: Diastolic blood pressure; SBP: Systolic blood pressure.

**Table 3:** Results of Working Memory Tasks.

	PD Control	HOA Control	PD Stress	HOA Stress
**Digit Span Forward** **TE-ML**	6.20 ± 0.94	6.60 ± 0.99	6.27 ± 1.34	6.40 ± 1.30
**Digit Span Backward** **TE-ML**	4.20 ± 1.15 ^b^	5.40 ± 1.35 ^b^	4.60 ± 1.30	5.00 ± 1.36
**Digit Span Forward** **RT 3 Digits(s)**	6.81 ± 2.36 ^bb^	4.65 ± 1.72 ^bb^	6.02 ± 2.08 ^b^	4.42 ± 1.15 ^b^
**Digit Span Forward** **RT 4 Digits(s)**	5.79 ± 1.24	5.11 ± 1.54 ^a^	5.88 ± 1.30 ^bbb^	4.25 ± 0.73 ^a,bbb^
**Digit Span Forward** **RT 5 Digits (s)**	6.48 ± 1.85	6.87 ± 1.81	6.32 ± 1.32	6.09 ± 3.19
**Digit Span Backward** **3 Digits (s)**	5.88 ± 2.25	4.77 ± 1.64	6.19 ± 2.00	5.86 ± 2.01
**Digit Span Backwards** **4 Digits (s)**	9.18 ± 3.80	8.26 ± 2.72	10.32 ± 5.51	9.03 ± 4.35

Means and standard deviations for all working memory measures for both groups and both conditions. For within subjects intervention comparisons ^a^ *p* < 0.05, For between subjects comparisons for a particular condition ^b^ *p* < 0.05, ^bb^ *p* < 0.01, ^bbb^ *p* < 0.001. HOA; healthy older adult; PD: persons with Parkinson’s disease; RT: Response Time; TE-ML Two Error-Maximum Length.

## Data Availability

The data supporting the findings of this pilot study are available on request from the corresponding author. The data are not publicly available due to ethical restrictions.
